# Exploring Molecular Mechanisms and Biomarkers in COPD: An Overview of Current Advancements and Perspectives

**DOI:** 10.3390/ijms25137347

**Published:** 2024-07-04

**Authors:** Chin-Ling Li, Shih-Feng Liu

**Affiliations:** 1 Department of Respiratory Therapy, Kaohsiung Chang Gung Memorial Hospital, Kaohsiung 833, Taiwan; musquito16@cgmh.org.tw; 2 Division of Pulmonary and Critical Care Medicine, Department of Internal Medicine, Kaohsiung Chang Gung Memorial Hospital, Kaohsiung 833, Taiwan; 3 College of Medicine, Chang Gung University, Taoyuan 333, Taiwan

**Keywords:** chronic obstructive pulmonary disease, molecular pathogenesis, molecular biomarkers, epigenetics and gene regulation, targeted therapies, precision medicine

## Abstract

Chronic obstructive pulmonary disease (COPD) plays a significant role in global morbidity and mortality rates, typified by progressive airflow restriction and lingering respiratory symptoms. Recent explorations in molecular biology have illuminated the complex mechanisms underpinning COPD pathogenesis, providing critical insights into disease progression, exacerbations, and potential therapeutic interventions. This review delivers a thorough examination of the latest progress in molecular research related to COPD, involving fundamental molecular pathways, biomarkers, therapeutic targets, and cutting-edge technologies. Key areas of focus include the roles of inflammation, oxidative stress, and protease–antiprotease imbalances, alongside genetic and epigenetic factors contributing to COPD susceptibility and heterogeneity. Additionally, advancements in omics technologies—such as genomics, transcriptomics, proteomics, and metabolomics—offer new avenues for comprehensive molecular profiling, aiding in the discovery of novel biomarkers and therapeutic targets. Comprehending the molecular foundation of COPD carries substantial potential for the creation of tailored treatment strategies and the enhancement of patient outcomes. By integrating molecular insights into clinical practice, there is a promising pathway towards personalized medicine approaches that can improve the diagnosis, treatment, and overall management of COPD, ultimately reducing its global burden.

## 1. Introduction

Chronic obstructive pulmonary disease (COPD) remains a significant global health burden, with an escalating prevalence, specifically among aging populations and areas with elevated incidences of smoking and air pollution [1]. The World Health Organization (WHO) anticipates that by 2030, COPD will rank as the third leading cause of mortality worldwide [2]. This progressive lung disease is characterized by consistent airflow limitations, typically associated with symptoms such as chronic coughing, sputum production, and shortness of breath during exertion. As patients’ conditions advance, they are likely to experience exacerbations leading to further declines in lung function and quality of life [3].

The ramifications of COPD extend beyond individual health, imposing substantial economic burdens on healthcare systems and society at large. The direct costs encompass hospital admissions, medication, and attendance at outpatient clinics [4], while indirect costs arise from productivity losses and disability, amplifying the socioeconomic impact. Furthermore, the association of COPD with comorbidities such as cardiovascular disease, osteoporosis, and depression complicates its management and adds to the pressures on healthcare provisions [5].

Against this backdrop, molecular research has emerged as a pivotal tool in decoding the complex pathogenesis of COPD. Until recently, the understanding of COPD was primarily centered around its correlation with cigarette smoking and environmental exposures. However, innovative advances in molecular biology have illuminated the complex interplay between genetic susceptibility, epigenetic modifications, and intricate cellular and molecular pathways in the development and progression of COPD [6].

Molecular investigations have underscored the critical role of inflammation, oxidative stress, and protease–antiprotease imbalance in driving the airway inflammation, remodeling, and parenchymal destruction observed in COPD [7]. Additionally, genetic studies have linked specific susceptibility loci and gene variants with COPD risk and severity, shedding light on the genetic underpinnings of disease heterogeneity and potential therapeutic targets [8].

Given this level of complexity, molecular research holds immense potential to guide the path towards personalized COPD management strategies. By delving deeper into the molecular bases of disease phenotypes and identifying biomarkers indicative of disease activity and treatment responses, it paves the way forward for targeted therapies and precision medicine tailored to individual patient profiles [9]. Consequently, understanding the role of molecular research in COPD is integral not just for advancing scientific knowledge, but for transforming these insights into meaningful advancements in patient care and outcomes [10].

## 2. Molecular Pathogenesis of COPD

COPD is characterized by two primary conditions: emphysema and chronic bronchitis. Emphysema involves the destruction of alveoli, leading to reduced surface area for gas exchange. Chronic bronchitis is marked by chronic inflammation of the bronchial tubes, resulting in excessive mucus production and airway obstruction.

COPD is characterized by an intricate interaction of molecular mechanisms that propel its development and progression. Understanding these underlying processes is crucial for pinpointing potential therapeutic targets and formulating personalized treatment methodologies [11].

### 2.1. Inflammation

A fundamental element in the pathogenesis of COPD is inflammation, engineering a chain of immune responses within the respiratory tract. Chronic exposure to harmful particulates, such as tobacco smoke and air pollution, triggers the activation of innate immune cells, including macrophages, neutrophils, and dendritic cells [12]. These immune cells release pro-inflammatory mediators, primarily tumor necrosis factor-alpha (TNF-α), interleukin-6 (IL-6), and interleukin-8 (IL-8), leading to enduring inflammation and tissue damage [13]. Moreover, the enlistment and activation of adaptive immune cells like T cells exacerbate the inflammatory milieu, contributing to airway remodeling and lung function deterioration [14].

### 2.2. Oxidative Stress

Oxidative stress, resulting from an imbalance of reactive oxygen species (ROS) production and antioxidant defenses, is a defining characteristic of COPD pathogenesis [15]. Cigarette smoke, COPD’s primary risk factor, is a potent source of oxidative stress, causing cellular damage and dysfunction in airway epithelial cells and pulmonary macrophages [16]. Oxidative damage mediated by ROS to lipids, proteins, and DNA enhances inflammation, impairs mucociliary clearance, and obstructs repair mechanisms within the lung parenchyma [17]. Furthermore, oxidative stress augments the protease–antiprotease imbalance by activating matrix metalloproteinases (MMPs) and inhibiting tissue inhibitors of metalloproteinases (TIMPs), causing extracellular matrix degradation and emphysematous changes [18].

### 2.3. Protease–Antiprotease Imbalance

A crucial pathogenic mechanism underlying COPD is the protease–antiprotease imbalance, characterized by excessive proteolytic activity and impaired protease inhibition within the lung microenvironment [19]. Serine proteases, including neutrophil elastase and proteinase-3, are discharged by activated inflammatory cells, leading to tissue damage by degrading extracellular matrix proteins and disrupting lung structure [20]. In contrast, endogenous antiproteases like alpha-1 antitrypsin (AAT) play a crucial role in countering protease activity and preserving tissue integrity. The genetic deficiency or functional impairment of AAT predisposes individuals to early-onset emphysema, emphasizing the role of protease–antiprotease balance in COPD susceptibility [21].

### 2.4. Genetic Susceptibility

Genetic factors significantly contribute to COPD susceptibility and phenotypic variability, with estimates of heritability ranging from 40% to 77% [22]. Genome-wide association studies (GWASs) have identified numerous genetic variants associated with COPD risk, encompassing genes involved in inflammatory pathways (e.g., IL-6, TNF), antioxidant defense mechanisms (e.g., glutathione S-transferase), and lung development (e.g., surfactant proteins) [23]. Additionally, epigenetic modifications such as DNA methylation and histone acetylation modulate gene expression patterns in response to environmental exposures, further influencing COPD susceptibility and disease progression [24].

### 2.5. Lung Tissue Remodeling

COPD is associated with lung tissue remodeling, including changes in the structure of the airways and alveoli. This remodeling involves the thickening of airway walls, destruction of alveolar attachments, and enlargement of air spaces (emphysema). These structural changes contribute to airflow limitation and reduced lung function.

The molecular pathogenesis of COPD is typified by a complex interaction of inflammation, oxidative stress, protease–antiprotease imbalance, and genetic susceptibility. Understanding these processes offers promise for identifying novel therapeutic targets and developing personalized treatment strategies to mitigate disease progression and improve clinical outcomes for COPD patients [25].

## 3. Molecular Biomarkers in COPD (Table 1)

Molecular biomarkers hold pivotal roles in COPD diagnosis, prognosis, and monitoring [9]. These biomarkers encompass a variety of molecules, including proteins, nucleic acids, and metabolites, that are identifiable in a range of biological specimens like blood, sputum, and imaging methodologies. Employing molecular biomarkers promises to enhance the precision of COPD diagnosis, stratify disease severity, predict exacerbations, and orchestrate therapeutic interventions [26].

### 3.1. Blood-Based Biomarkers

A minimally invasive strategy for examining systemic inflammation, oxidative stress, and molecular signatures associated with COPD pathogenesis is provided by blood-based biomarkers [27]. Inflammatory biomarkers, like C-reactive protein (CRP), fibrinogen, and white blood cell counts, reflect systemic inflammation and are linked to disease severity and exacerbation risks [28]. Additional biomarkers of oxidative stress, including malondialdehyde (MDA) and oxidized DNA/RNA products, offer insights into systemic redox imbalances observed in COPD [29]. Furthermore, circulating biomarkers indicative of extracellular matrix remodeling (e.g., MMPs, TIMPs) and endothelial dysfunction (e.g., endothelin-1, von Willebrand factor) may provide prognostic indicators for disease progression and cardiovascular comorbidities in COPD patients [30].

**Table 1:** COPD biomarkers in blood.

Biomarker	Function	Clinical Relevance	References
C-reactive protein	Indicator of systemic inflammation	Linked to disease severity and exacerbation risks	[28]
Fibrinogen	Role in coagulation cascade	Associated with cardiovascular comorbidities	[31]
White blood cells	Immune response indicator	Reflects inflammation levels and disease progression	[32]
Malondialdehyde	Marker of oxidative stress	Reflects systemic redox imbalances in COPD	[33]
MMPs	Proteins involved in tissue	Extracellular matrix remodeling and lung function	[30]

### 3.2. Sputum-Based Biomarkers

Sputum-based biomarkers offer a more direct method to inspect the airway inflammation and cellular composition characteristic of COPD [34]. Induced sputum analysis allows the quantification of inflammatory cells (e.g., neutrophils, eosinophils), inflammatory mediators (e.g., cytokines, chemokines), and protease activity (e.g., neutrophil elastase) within the airway microenvironment [35]. Elevated sputum eosinophils are linked to increased exacerbation risk and corticosteroid responsiveness in COPD patients, marking their usefulness as predictive biomarkers for treatment selection. Moreover, sputum proteomic and metabolomic profiling shows potential for identifying novel molecular signatures associated with COPD phenotypes and response to therapy [36].

### 3.3. Imaging Biomarkers

Non-invasive tools to assess structural and functional abnormalities within the respiratory system and monitor COPD disease progression are provided by imaging biomarkers [37]. Techniques such as computed tomography (CT) imaging enable the quantification of emphysema severity, airway wall thickness, and pulmonary vascular remodeling—facilitating disease staging and phenotypic characterization. Functional imaging methods, notably positron emission tomography (PET) and magnetic resonance imaging (MRI), offer insights into regional lung perfusion, ventilation, and inflammation, thereby providing crucial information for treatment planning and monitoring therapy response [38].

Expanding the use of molecular biomarkers into clinical practice can ultimately lead to precision medicine in COPD management. By utilizing these biomarkers for patient stratification, treatment selection, and disease progression monitoring, clinicians may optimize therapeutic interventions and enhance COPD patient outcomes [39]. Nevertheless, the additional validation and standardization of biomarker assays are required to adopt these strategies routinely and ensure their effectiveness and reliability in clinical practice.

## 4. Therapeutic Targets and Personalized Medicine in COPD (Table 2)

A paradigm shift in the handling of COPD is being observed by means of molecularly targeted therapies and precision medicine techniques. They provide room for personalized treatment strategies synced with individual patient profiles. Targeting these specific pathways associated with COPD pathogenesis helps modify disease progression, minimize exacerbation events, elevate respiratory conditions, and enhance life quality [40].

**Table 2:** COPD therapeutic targets.

Therapeutic Target	Mechanism	Potential Clinical Impact	References
PDE4 inhibitors	Anti-inflammatory agent	Reduced exacerbation rates and improved lung function	[41,42]
IL-5 monoclonal antibodies	Targeting eosinophilic inflammation	Reduced exacerbation frequency and enhanced lung function	[43]
M3 receptor antagonists	Improved bronchodilation	Potency and safety profiles surpassing traditional therapies	[44,45]
Biologic agents targeting IL-17 pathway	Modulating immune responses	Potential for treating neutrophilic inflammation	[46]
Beta-2 adrenergic receptor	Improved bronchodilation	Long-established, symptomatic, and effective therapeutic target	[47]

### 4.1. Molecularly Targeted Therapies

Molecularly targeted therapies aim to tackle the molecular mechanisms facilitating disease progression, moving beyond traditional bronchodilators and anti-inflammatory agents used in COPD treatment. For instance, phosphodiesterase-4 (PDE4) inhibitors like roflumilast are used to prevent the degradation of cyclic adenosine monophosphate (cAMP) within inflammatory cells, thereby functioning as an anti-inflammatory agent. The effects of roflumilast have been seen as reduced exacerbation rates along with improved lung function in severe COPD patients with chronic bronchitis [41].

COPD also sees potential in pathways such as interleukin-5 (IL-5), crucial in eosinophilic inflammation and airway remodeling. Monoclonal antibodies targeting IL-5—mepolizumab and benralizumab—have proved to be competent enough in reducing exacerbation frequency and enhancing lung function for patients with eosinophilic COPD. For patients with neutrophilic inflammation and airway remodeling, targeting the interleukin-17 (IL-17) pathway with monoclonal antibodies like secukinumab shows promise [43].

Innovations in bronchodilators targeting certain muscarinic receptor subtypes have been noted (e.g., M3 receptor antagonists), along with β2-adrenergic receptor agonists with an extended duration of action (e.g., ultra-LABAs). These are viewed as worthy contenders, having potency and safety profiles possibly surpassing old-style bronchodilator therapies [44].

### 4.2. Precision Medicine Approaches

Precision medicine works on recognizing distinct COPD subtypes/endotypes relying on molecular mechanisms, clinical phenotypes, and biomarker profiles. This helps doctors to provide tailored treatment strategies for individual patients. One way is COPD classification based on the inflammation phenotype, with different responses to therapy noticed in eosinophilic and neutrophilic subtypes [48].

Treatment procedures can also be directed by a biomarker-oriented treatment algorithm—like the blood eosinophil count—by recognizing patients most likely to profit from particular therapies such as inhaled corticosteroids (ICSs) or biologic agents addressing eosinophilic inflammation. In addition, genetic data usage like alpha-1 antitrypsin (AAT) deficiency status in treatment strategies allows a personalized approach to managing COPD, such as augmentation therapy with AAT replacement for AAT-deficient individuals [49].

Emerging “omics” technologies, namely genomics, transcriptomics, and metabolomics, extend potential opportunities for discovering molecular signatures relevant to treatment response and COPD progression. Multi-omics data usage helps researchers identify novel therapeutic targets and build predictive models to individualize treatment methods based on patient-specific attributes and disease trajectories [50].

In summary, we see promising strategies for optimizing COPD management and augmenting patient outcomes in molecularly targeted therapies as well as precision medicine procedures. Clinicians’ ability to recognize and address specific molecular pathways implicated in COPD pathogenesis equips them to fit treatment procedures to individual patient profiles, maximizing therapeutic efficacy while reducing adverse effects to a minimum. However, biomarker validation, treatment algorithm refinement, and molecular insights’ translation into clinical practice requires continued research to fully harness personalized medicine’s potential in COPD care [51].

## 5. Epigenetics and Gene Regulation in COPD

**Figure 1:**
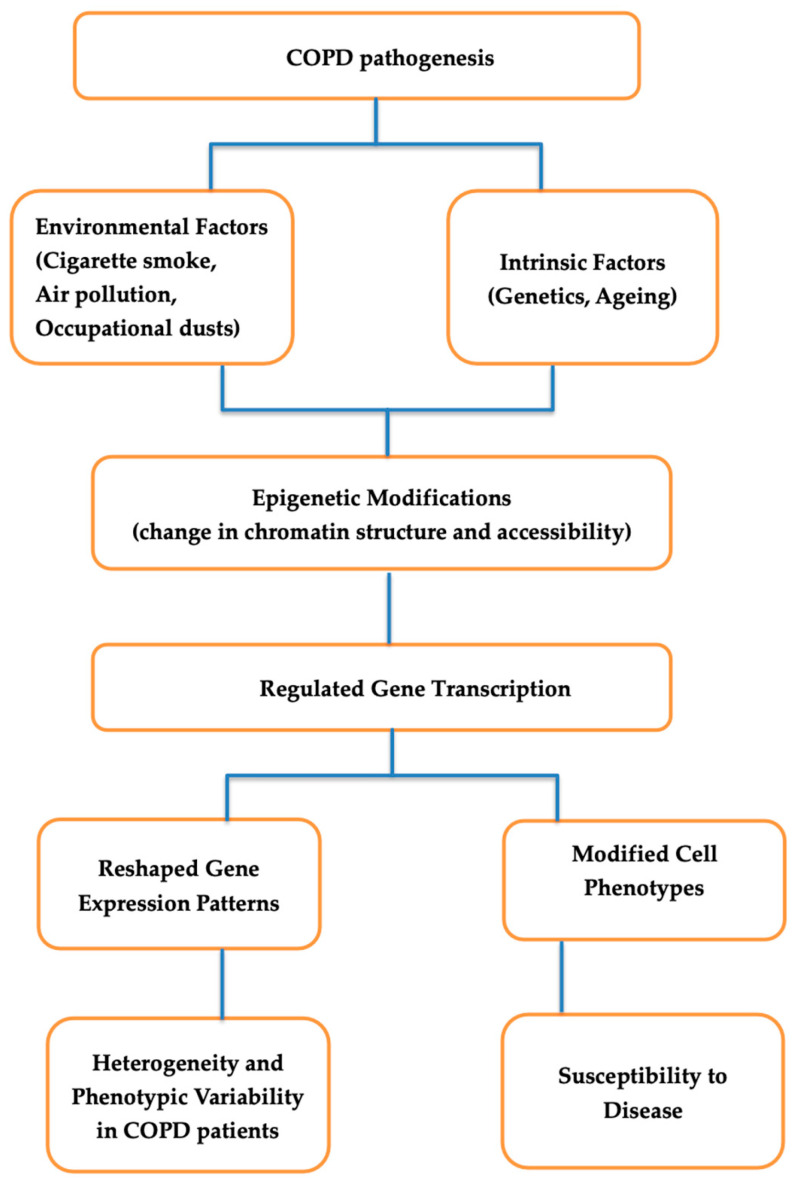
The flow of factors contributing to COPD pathogenesis.

Epigenetic modifications and gene regulatory networks significantly affect the pathogenesis of COPD, reshaping gene expression patterns, cell phenotypes, and susceptibility to the disease. The modification of chromatin structure and accessibility through epigenetic mechanisms provides regulated gene transcription in response to environmental exposures and intrinsic factors, thus contributing to the heterogeneity and phenotypic variability seen in COPD patients [52]. This representation summarizes the flow of factors contributing to COPD pathogenesis, starting from environmental and intrinsic factors, leading to epigenetic modifications, regulated gene transcription, reshaped gene expression patterns, modified cell phenotypes, and ultimately impacting susceptibility to disease and heterogeneity in COPD patients (Figure 1).

### 5.1. Epigenetic Modifications

Epigenetic modifications are a diverse collection of reversible chemical alterations to DNA and histone proteins, including DNA methylation, histone acetylation, and non-coding RNA-mediated regulation. Abnormal epigenetic patterns can contribute to the pathogenesis of COPD, reflecting the combined effects of environmental exposures (e.g., cigarette smoke, air pollution) and genetic predisposition [53].

DNA methylation involves the addition of methyl groups to cytosine residues within CpG dinucleotides, commonly associated with transcriptional repression in gene promoter regions. Widespread alterations in DNA methylation patterns have been observed in COPD patients, affecting the regulation of genes tied to inflammatory pathways (e.g., TNF, IL-6), antioxidant defense mechanisms (e.g., GSTP1), and extracellular matrix remodeling (e.g., MMP9) [54]. Histone modifications dynamically regulate chromatin structure and gene transcription by modulating histone–DNA interactions and guiding transcriptional regulators. In response to oxidative stress and inflammatory signaling, changes in histone acetylation patterns occur in COPD [55].

### 5.2. Gene Regulatory Networks

Gene regulatory networks manage complex interactions among transcription factors, non-coding RNAs, and epigenetic modifiers to orchestrate gene expression affecting COPD pathogenesis. The disruption of these networks in COPD patients can lead to dysregulated inflammatory responses, impaired tissue repair mechanisms, and increased susceptibility to environmental insults [56]. 

Transcription factors including nuclear factor-kappa B (NF-κB), activator protein-1 (AP-1), and hypoxia-inducible factor-1α (HIF-1α) play crucial roles mediating inflammatory signaling and cellular responses to hypoxic stress in COPD. The dysregulation of these transcriptional regulators results in the prolonged activation of pro-inflammatory genes, exacerbating airway inflammation and tissue damage in COPD patients [57].

### 5.3. Implications for Understanding Disease Heterogeneity and Identifying Therapeutic Targets

Epigenetic modifications and gene regulatory networks contribute to the disease heterogeneity in COPD by shaping individual susceptibility to environmental exposures, modulating inflammatory phenotypes, and influencing treatment responses. These findings hold promise for identifying novel therapeutic targets and developing personalized treatment strategies for COPD patients [58].

In conclusion, epigenetic modifications and gene regulatory networks play a central role in COPD pathogenesis, influencing disease heterogeneity and providing insights into novel therapeutic targets. Further research in this area can advance precision medicine approaches tailored to individual patient profiles and improve outcomes for individuals with COPD [59].

## 6. Emerging Technologies and Omics Approaches in COPD Research

The genesis of omics technologies has significantly transformed our comprehension of multifaceted diseases like COPD by granting the comprehensive molecular profiling of biological systems across various levels, ranging from DNA, RNA, and proteins to metabolites [60]. These omics methodologies offer a comprehensive view of disease pathogenesis, facilitate the identification of novel biomarkers, and demystify potential therapeutic targets, thereby driving rapid progress in COPD research and its clinical management.

### 6.1. Genomics

Genomics entails a complete study of an organism’s entire DNA sequence, inclusive of variations in the genome related to disease susceptibility and progression. Genome-wide association studies (GWASs) have spotlighted genetic loci linked to COPD risk and severity, underlining the role of both common and rare genetic variants in contributing to disease heterogeneity [61]. Furthermore, the application of next-generation sequencing (NGS) technologies has enabled the identification of rare variants, copy number variations (CNVs), and structural rearrangements instrumental in COPD evolution, offering valuable insights into genetic mechanisms that underscore disease susceptibility and treatment responsiveness [62].

### 6.2. Transcriptomics

Transcriptomics emphasizes analyzing a complete set of RNA transcripts (transcriptome) produced by cells or tissues under specific circumstances. COPD-focused transcriptomic studies have elucidated gene expression patterns tied to disease phenotypes, inflammation responses, and therapeutic interjections. Differential gene expression analysis has identified dysregulated pathways—such as oxidative stress, inflammatory signaling, and extracellular matrix remodeling—thereby providing mechanistic insights into COPD pathogenesis and progression [63]. Moreover, RNA sequencing (RNA-seq) has aided in identifying alternative splicing events, non-coding RNAs, and gene isoforms crucial to COPD biology and therapeutic targeting [64].

### 6.3. Proteomics

Proteomics involves a comprehensive analysis of proteins expressed within cells, tissues, or biological fluids, providing critical insights into protein abundance, post-translational modifications, and protein–protein interactions in health and disease states. Proteomic profiling in COPD has divulged alterations in protein expression profiles related to airway inflammation, oxidative stress, and tissue remodeling [65]. Mass spectrometry-based approaches have facilitated the identification and quantification of proteins involved in disease pathogenesis, biomarker discovery, and drug target validation [66]. Furthermore, targeted proteomics assays like multiple reaction monitoring (MRM) and selected reaction monitoring (SRM) offer the sensitive and specific detection of protein biomarkers in clinical samples, aiding their transformation into standard diagnostic assays [67].

### 6.4. Metabolomics

Metabolomics focuses on analyzing a complete set of small-molecule metabolites (metabolome) present in cells, tissues, and biological fluids, thereby reflecting cellular metabolism and physiological states. Metabolomic profiling in COPD has flagged disturbances in metabolic pathways related to oxidative stress, energy metabolism, and lipid homeostasis [68]. Techniques like gas chromatography–mass spectrometry (GC-MS) and liquid chromatography–mass spectrometry (LC-MS) have paved the way for identifying and quantifying metabolites relevant to COPD pathogenesis and disease progression [69]. Metabolomic biomarkers such as oxidative stress markers, lipid mediators, and amino acid derivatives show promise in predicting disease outcomes, monitoring treatment response, and stratifying COPD patients based on metabolic phenotypes [33].

### 6.5. Integration of Multi-Omics Data

The integration of data from multiple omics platforms—inclusive of genomics, transcriptomics, proteomics, and metabolomics—provides a comprehensive understanding of molecular mechanisms underlying COPD pathogenesis and heterogeneity. Multi-omics approaches aid in identifying molecular signatures tied to disease subtypes, progression trajectories, and treatment responses, thereby guiding personalized therapeutic interventions and precision medicine approaches [70]. Moreover, systems biology and bioinformatics tools facilitate the integration and analysis of omics datasets, uncovering complex interactions and regulatory networks propelling COPD biology. By combining insights from genomics, transcriptomics, proteomics, and metabolomics, researchers can unearth novel biomarkers, therapeutic targets, and diagnostic tools for ameliorating COPD management and propelling personalized medicine [71].

## 7. Challenges and Future Directions in COPD Research

### 7.1. Translating Molecular Research into Clinical Applications

Despite significant advancements in molecular research, translating these findings into clinically actionable interventions remains a challenge in COPD management. One major hurdle is the complex and multifactorial nature of COPD, which necessitates a comprehensive understanding of disease mechanisms and personalized treatment strategies [72]. 

Additionally, variability in patient responses to therapy, heterogeneity in disease phenotypes, and limitations in biomarker validation add to the complexities of implementing molecularly targeted therapies and precision medicine approaches into routine clinical practice [73]. 

To address these challenges, interdisciplinary collaboration among clinicians, researchers, and industry partners is critical to bridge the gap between bench and bedside [74]. Robust clinical trials incorporating molecular biomarkers and patient stratification strategies are needed to evaluate the safety and efficacy of targeted therapies in specific COPD subgroups [75]. Additionally, the standardization of protocols for biomarker validation and regulatory approval processes is essential to ensure the reliability and reproducibility of molecular diagnostic assays and therapeutic interventions [76].

### 7.2. Future Perspectives on Targeted Therapies and Precision Medicine

Despite existing challenges, the future of COPD research holds promise for the development of novel targeted therapies and precision medicine approaches tailored to patient-specific treatment profiles. Advances in omics technologies, computational biology, and systems medicine present unprecedented opportunities for identifying disease-specific biomarkers, deciphering associated molecular pathways, and predicting treatment responses in COPD [77].

One potential research direction is the integration of multi-omics datasets to unravel complex interactions and regulatory networks underlying COPD pathogenesis [78]. This integrative approach can pave the way for personalized therapeutic interventions by identifying molecular signatures associated with disease subtypes, progression trajectories, and response to treatment. Moreover, advancements in drug discovery and development, including the repurposing of existing drugs and the design of newer targeted therapeutics, could provide avenues to address the current gaps in COPD management [79]. The promise of small-molecule inhibitors, targeting specific molecular pathways (e.g., inflammation, oxidative stress, protease activity [80]), and biologic agents [81] modulating immune responses or tissue repair mechanisms could revolutionize the clinical outcomes for COPD patients.

The advent of digital health technologies, wearable devices, and telemedicine platforms have given rise to possibilities for remote patient monitoring, real-time data collection, and personalized care delivery in COPD management [82]. Leveraging these technologies can lend clinicians the means to track disease progression, monitor treatment adherence, and empower patients for self-management purposes. At its full potential, this can optimize therapeutic interventions and potentially improve patient outcomes in COPD [83].

It is clear that addressing the challenges and embracing future directions in COPD research requires collaborative efforts, innovative approaches, and a patient-centric health strategy. Capitalizing on molecular research, targeted therapies, and digital health technologies can advance our understanding of the COPD pathogenesis [84] and pioneer clinical practice to optimize outcomes for individuals living with this debilitating respiratory condition.

## 8. Conclusions

Molecular research, in recent years, has made significant strides to enhance our understanding of the pathogenesis, progression, and heterogeneity of COPD [85]. By investigating molecular mechanisms, biomarkers, and therapeutic targets, researchers have gleaned valuable insights which hold great promise for improving both the management and clinical outcomes of COPD patients.

Key insights derived from molecular research, such as identifying inflammatory pathways, outlining oxidative stress mechanisms, highlighting protease–antiprotease imbalances, and discerning genetic predispositions, have not only heightened our understanding of the biology of COPD, but also paved the way for the breakthrough development of targeted therapies and precision medicine interventions tailored professionally to individual patient profiles [86]. The reverberations of molecular research for COPD management are wide-ranging. Molecular research, by illuminating disease mechanisms and identifying biomarkers indicative of disease severity, progression, and treatment responsiveness, facilitates clinicians in patient stratification, personalizing treatment regimens, and optimizing therapeutic outcomes [87]. Moreover, by integrating multi-omics datasets, researchers can unlock the potential for comprehensive disease characterization, predictive modeling, and the discovery of novel therapeutic targets, thus driving advancements in precision medicine for COPD [88].

However, maximizing the full potential of molecular research in COPD management demands continued investment, collaboration, and innovative advancements. Persistent investigative efforts are crucial to further explore the molecular foundation of COPD, validate crucial biomarkers, and transform scientific discoveries into clinically effective interventions [51].

Moreover, fostering interdisciplinary collaborations among academia, industry, and healthcare providers is an essential catalyst in the accelerated development and implementation of molecularly targeted therapies and precision medicine techniques into routine clinical practice [89].

In view of the global burden of COPD, along with the unfulfilled needs of patients, the urgency for sustaining funding and support for molecular research initiatives cannot be understated. Policymakers, funding agencies, and healthcare stakeholders can galvanize innovation, drive scientific progression, and ultimately improve the lives of millions affected by this chronic respiratory affliction by investing in and prioritizing COPD-focused research [90]. 

In a nutshell, molecular research bears tremendous potential to transform our understanding and subsequent treatment of COPD. By leveraging the power of molecular insights, personalized medicine, and collaborative partnerships, we stand at the cusp of a radical transformation in the management landscape of COPD, potentially lessening the burden of this devastating disease [91].

## Data Availability

No new data were created in this study. Data sharing is not applicable to this article.
